# Advances in Normobaric Hyperoxia Brain Protection in Experimental Stroke

**DOI:** 10.3389/fneur.2020.00050

**Published:** 2020-01-31

**Authors:** Zhiying Chen, Yuchuan Ding, Xunming Ji, Ran Meng

**Affiliations:** ^1^Department of Neurology, Xuanwu Hospital, Capital Medical University, Beijing, China; ^2^Department of Neurosurgery, School of Medicine, Wayne State University, Detroit, MI, United States; ^3^Department of Neurosurgery, Xuanwu Hospital, Capital Medical University, Beijing, China; ^4^Center of Stroke, Beijing Institute for Brain Disorders, Beijing, China

**Keywords:** normobaric hyperoxia, experimental stroke, brain trauma, protection, hyperbaric oxygen

## Abstract

As we all know that stroke is still a leading cause of death and acquired disability. Etiological treatment and brain protection are equally important. This review aimed to summarize the advance of normobaric-hyperoxia (NBHO) on brain protection in the setting of experimental stroke and brain trauma. We analyzed the data from relevant studies published on PubMed Central (PMC) and EMBASE, about NBHO on brain protection in the setting of experimental ischemic and hemorrhagic strokes and brain trauma, which revealed that NBHO had important value on improving hypoxia and attenuating ischemia damage. The mechanisms of NBHO involved increasing the content of oxygen in brain tissues, restoring the function of mitochondria, enhancing the metabolism of neurons, alleviating blood-brain barrier (BBB) damage, weakening brain cell edema, reducing intracranial pressure, and improving cerebral blood flow, especially in the surrounding of injured area of the brain, to make the neurons in penumbral area alive. Compared to hyperbaric oxygen (HBO), NBHO is more safe and more easily to transform to clinical use, whereby, further studies about the safety and efficacy as well as the proper treatment protocol of NBHO on human may be still needed.

## Introduction

The benefits of oxygen therapy in neuroprotection are unproven, with mechanisms of action still unclear (1, 2). This review focuses on the translational potential of normobaric therapy for ischemic protection.

Hypoxia is known to result in edema, apoptosis, and the death of nerve cells. Low oxygen environments cause leukocytes to infiltrate into hypoxic tissue, leading to tumor necrosis factor elevation, oxygen free radicals all resulting in an inflammatory reaction (3, 4). To date, there is no standardized normobaric oxygen therapy which has been adopted for improving hypoxic pathological changes in patients with brain damage.

In this review, we have analyzed data from relevant studies published in PubMed Central (PMC) and EMBASE, relating to normobaric-hyperoxia (NBHO) and brain protection in the setting of experimental ischemic and hemorrhagic strokes and brain trauma (5–8).

Herein, we summarize the experimental data, and the mechanisms of NBHO in acute stroke to create a go-to resource for future study.

## Concept And Application Of NBHO

The common oxygen therapies include hyperbaric oxygen (HBO) and NBHO. Hyperbaric oxygen (HBO) refers to administering high concentrations of oxygen in an environment where ambient pressure is greater than atmospheric pressure (9, 10). HBO is usually utilized for correcting hypoxia in disease entities such as carbon monoxide poisoning or wound healing. HBO use is limited by the need for a hyperbaric oxygen chamber, equipment, as well as some contraindications. This therapy is inconvenient to patients and difficult to employ in a post stroke emergent environment.

NBHO (high-flow oxygen or high-concentration oxygen) refers to the method of continuously administering oxygen to patients via mask or oxygen hood under normal atmospheric pressure. The commonly utilized oxygen concentration used is 40–100% (11, 12).

Compared to HBO, the advantages of NBHO are convenience, cost and safety. NBHO can be performed anywhere, even in the acute stroke setting. NBHO can reduce the infarcted volume and prolong the time window of thrombolytic treatment in ischemic stroke (13). In addition, it may protect brain tissues surrounding the hematoma in hemorrhagic stroke (5, 8, 12).

## Controversy In NBHO Use

Results from previous NBHO studies have been controversial. The role of NBHO in brain protection is mainly to improve brain metabolism and increase the tolerance of cells to damage (11, 14). The predominant accepted molecular mechanism involved is to inhibit the expression of HIF-1α, MMP-9, and Caspase-3 as well as to increase the expression of VEGF, ROS (3, 5, 6, 8, 12).

Other studies have shown that NBHO does not improve the hypoxic area after cerebral infarction or hemorrhage. Some have concluded that too much oxygen use might increase oxygen free radicals and lung injury (15, 16).

**Figure 1:**
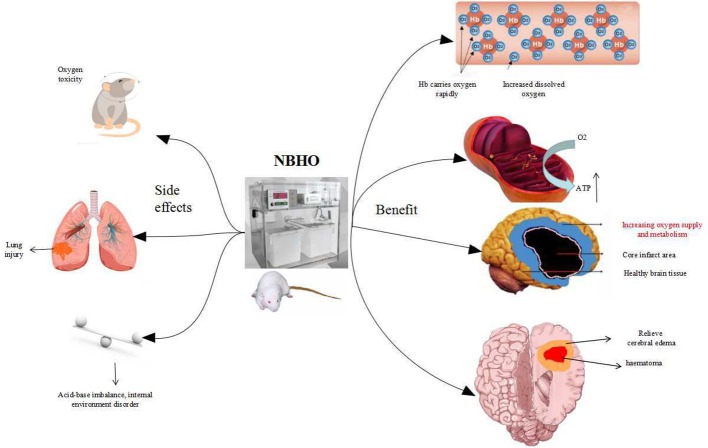
The benefits and adverse effects of NBHO.

NBHO protocols from numerous studies were reviewed and no clear protocol noted. Differences in starting time, frequency, concentration of oxygen, and the cycles of NBHO treatment were noted. Systemic conditions can also affect the outcomes of NBHO, such as glucose and other nutritional supply deficiencies. Poor ventilation and anemia can cause difficulty with oxygenation and render this therapy inadequate (4). Figure 1 displays a schematic diagram regarding the benefits and adverse effects of NBHO confirmed by existing animal studies.

## NBHO In Hemorrhagic Stroke

Local neurons surrounding the hemorrhagic lesion are in a hypoperfused state, related to the hematoma compression-mediated arterial perfusion decrease and venous outflow insufficiency. This results in mitochondrial dysfunction in affected cells, causing further cascading damage, all of which can have detrimental outcomes on function (17–19).

Previous studies have identified that blood pressure and body temperature control as well as that anti-oxygen free radical agent use could attenuate brain edema (20). However, the contribution of NBHO on improving the status of ischemia and hypoxia around a hematoma cavity is still not clear.

**Figure 2:**
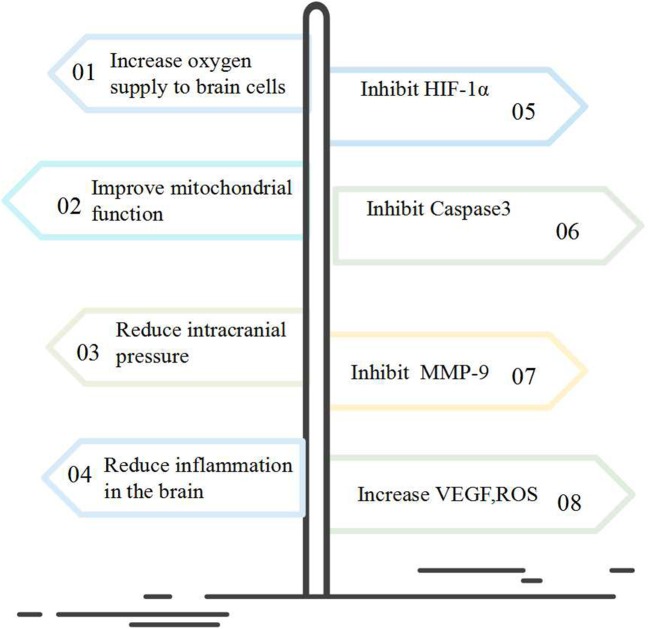
Possible mechanisms of NBHO in brain protection.

An animal study assessing NBHO therapy with oxygen delivered at a 90% concentration resulted in remarkable neuro-protection in rats with cerebral hemorrhage (ICH) (8). In this study, the ICH rats were treated by NBHO 6 h daily for three consecutive days, with the oxygen concentrations of 35, 50, or 90%, respectively. Compared to a non-treated ICH control group, NBHO-treated group revealed neurological functional improvement as well as brain edema attenuation, and HIF-1α and VEGF expression decline (Figure 2). Fewer apoptotic cells in the hematoma area at were also noted at 72 h post-ICH.

Research by Zhou et al. depicted the relationship between early NBHO therapy after ICH, blood-brain barrier damage and cerebral edema (21). This study revealed that NBHO for cerebral hemorrhage was effective within 30 min post-ICH; however, after more than 1 h, the intervention was invalid and may even cause free radical damage. In addition, this study proposed that the molecular mechanism of NBHO therapy on brain protection might involve the inhibition of MMP-9 and occludin. The above studies suggested that NBHO as a single factor contributed to neuro-protection in hemorrhagic stroke in addition to alleviating cerebral edema and maintaining the blood-brain barrier integrity (BBB).

A study by Norio Fujiwara et al. applied 100% of NBHO per-hour to 2 h in rats after ICH. Although the arterial oxygen concentration increased, NBHO did not affect any of the functional outcomes (22). The authors proposed that the no-protective effect might be a result of insufficient duration of NBHO treatment. Furthermore, 100% of oxygen may not be the most suitable concentration in NBHO treatment. The optimal concentration will need further study.

## NBHO In Ischemic Stroke

Blood flow interruption and hypoxia are the main causes of cell death after ischemic stroke. Thrombolysis and mechanical thrombectomy are time limited therapies (23). The pressure of oxygen in brain tissue is highly significant in the treatment of acute ischemic stroke.

A previous study revealed that early NBHO use attenuated BBB damage and improved the outcome of stroke treated with delayed rtPA. The authors concluded NBHO might be an effective adjunctive agent in extending the time window of rtPA thrombolysis (24). NHBO use can not only improve the anoxic metabolism in acute stroke, but also prevent the damage of blood-brain barrier in late stages. The probable mechanism may be that NBHO inhibits Matrix metalloproteinase-9 and tight junction protein degradation (24).

Research by Ding et al. demonstrated that NBHO started in the acute stage inhibited lactic acidosis in transient and permanent ischemic stroke (25). NBHO started 2 h after ischemic stroke, with 95% oxygen was given for 6 h. The results suggested that the protective effects of NBHO persisted longer than 2 weeks. In another experiment, Ding et al. found that NBHO therapy had the potential to reduce the level of blood occludins, protect the BBB and improve outcomes in patients with acute ischemic stroke undergoing intravenous rtPA thrombolysis (13).

Meanwhile, a study by Jean-Claude Baron suggested that NBHO alleviated brain damage in rats with transient cerebral ischemia by preventing neuronal death and microglial inflammation, as well as sensorimotor impairment (26).

Conversely, Elmira Pasban showed that early NBHO use did not reduce ischemic brain injuries and also did not produce protective effects against vasogenic cerebral edema formation or BBB disruption following acute ischemic stroke (27, 28). The MCAO model was used for infarction for 90 min and reperfusion lasted for 24 h. The NBHO protocol used 95% oxygen and 5% CO_2_ at 3 L/min. NBHO started at the 15th minute after MCAO and lasted for 90 min. We consider that the negative result of the NBHO was that the intervention time was too short and there was no continuous multi-day oxygen supply.

The protective role of NBHO in acute cerebral infarction had also been studied in clinical cases. Different studies have concluded with various results. In cerebral infarction animal model, NBHO protected the BBB by improving cerebral perfusion and oxygen metabolism, inhibiting of MMP-9, AQP-4, and NHE1 expression and reducing of TJP degradation. This may be a plausible molecular mechanism of protection (5, 6, 8, 24, 26). Standard NBHO protocols have not been established in the clinical arena.

## NBHO In Brain Injury

In patients with cranio-cerebral trauma, secondary injury refers to cerebral ischemia (cerebral hypoperfusion) caused by hypoxia, hypotension, intracranial hypertension, and cerebral vasospasm. Among them, cerebral ischemia and hypoxia are the most common pathological changes in secondary injury and are important prognostic factors after brain trauma (29, 30). In one study 100% NBHO at 2 h after brain trauma for 4 h twice a day resulted in increased mitochondrial matrix metalloprotease, reduced mitochondrial malondialdehyde production, reversed mitochondrial MMP, improved mitochondrial function and structure, and reduced apoptosis and apoptosis-related Caspase3 protein expression (31).

Use of micro-dialysis technology to detect the efficacy of early NBHO therapy in patients with brain trauma has shown increased levels of glucose and decreased lactic acid in cerebrospinal fluid resulting in the decline of the ratios of lactic acid to glucose and lactic acid to pyruvate. This signifies NBHO could improve the redox state of the brain (32, 33). Moreover, the above studies showed that NBHO could reduce the intracranial pressure in patients with brain trauma (32–34).

Conversely, Talley et al. demonstrated that NBHO increased the volume of brain damage in a rat model with a moderate traumatic brain injury (TBI), in which 0.5–3 h of 100% oxygen was administered after TBI (35, 36). The NBHO group did not improve cerebral blood flow in contused brain tissue or neurological function scores at 7 and 14 days. In addition, there was insufficient brain perfusion caused by reactive oxygen species (ROS) and vasoconstriction. One hundred percent oxygen therapy was used in this study. Further studies adjusting the concentration of O_2_, altering the timing, or extending the duration of NBHO exposure along with varying the range of injury severity will be necessary to improve the understanding of the potential benefits or limitations of NBHO therapy after TBI.

## Adverse Effects Of NBHO

NBHO use may have some adverse effects such as oxygen poisoning, lung injury and brain excito-toxicity.

Study by Westra et al. reported that immediate pretreatment with either hyperbaric (2.5 ATM for 1 h) or NBHO (100% FiO_2_ for 1 h) increased brain edema and worsened neurological function at 24 h following TBI (37). Additionally, Quintard et al. found that NBHO increased the amount of glutamate in the brain in patients with severe TBI, leading to brain excito-toxicity and aggravation of secondary brain damage. TBI is often associated with epilepsy, which can increase ROS and glutamate and decrease gamma-aminobutyric acid. This can cause decreased oxygen concentration in brain tissue during seizures (36). In addition, as the eyes, heart, digestive tract, and other organs are sensitive to high concentration of oxygen, NBHO may cause symptoms such as lens degeneration, angina, nausea and vomiting (38, 39).

In order to reduce the adverse effects, NBHO should be given by intermittent high concentrations model with 41–90% of oxygen, and blood acid-base balance and oxygen and carbon dioxide concentrations should be monitored regularly to prevent oxygen poisoning. If necessary, antioxidant drugs such as vitamin C and vitamin E should be administered. It is best to use a venturi mask or HHFNC (Humidified high-flow nasal cannula) in acute stroke patients with chronic obstructive pulmonary disease. Ordinary masks and oxygen storage masks are not recommended. CO_2_ retention risk factors can be considered to evaluate the ESCAPE principle. The ESCAPE principles for the assessment of CO_2_ retention risk factors include: bronchi ectasia, spinal disease, chest disease, airway obstructed disease, paralysis, elevated body weight (40). High-risk patients are not recommended to use oxygen therapy (41, 42).

Current research is focused on improving oxygen supply and metabolism in brain tissue. There are few reports on the effects of NBHO on excitatory amino acids and intracellular calcium ion concentrations. Further monitoring of potential adverse effects related to NBHO will be necessary in the future.

## Future Prospects

To sum up, standard NBHO appears to be a promising potential neurotherapeutic. The molecular mechanism of NBHO may inhibit the expression of HIF-1α, MMP-9, Caspase-3, and increase the expression of VEGF, ROS, etc., thereby increasing the utilization of oxygen molecules by cells (Figure 2).

NBHO's easy use allows it to be performed anywhere with a mask or nasal catheter. No special requirements for the treatment environment are necessary. This therapy is inexpensive and can be administered in remote areas.

**Figure 3:**
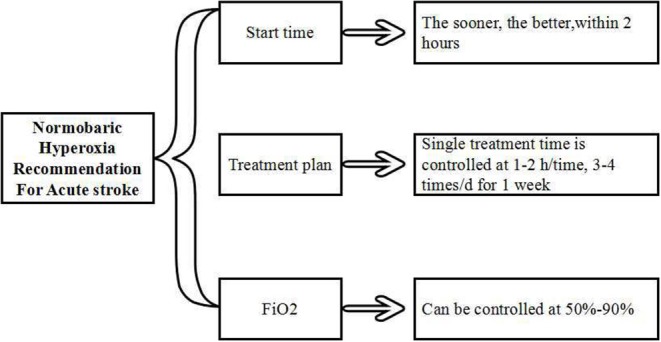
Protocol of NBHO use in stroke.

All studies mentioned above revealed that the initial oxygenation time was as early as possible, especially within 2 h after stroke. The ideal protocol for this appears to be controlled at 1-2 h/time, 3–4 times/d for 1 week, FiO_2_ could be controlled at 50–90% (Figure 3). Further study and clinical randomized controlled trials are still needed in the future to better characterize this therapy.

## Author Contributions

RM: manuscript drafting and revision, and study concept and design. ZC: manuscript drafting and revision, study concept and design, collection, assembly, and interpretation of the study. YD and XJ: deeply edited the revised version and contributed critical revision.

### Conflict of Interest

The authors declare that the research was conducted in the absence of any commercial or financial relationships that could be construed as a potential conflict of interest.

## Abbreviations

NBHO: normobaric hyperoxia
HBO: Hyperbaric oxygen
ICH: intracerebral hemorrhage
BBB: Blood-brain barrier
AIS: Acute ischemic stroke
tPA: tissue-type plasminogen activator
TBI: traumatic brain injury
FiO_2_: Fraction of inspiration O_2_
TJP: tight junction proteins
MMP-9: Matrix metalloproteinase-9
HIF-1α: hypoxia-inducible factor-1α
VEGF: vascular endothelial growth factor
CO_2_: carbon dioxide.
